# Spousal violence in sub-Saharan Africa: does household poverty-wealth matter?

**DOI:** 10.1186/1742-4755-11-45

**Published:** 2014-06-17

**Authors:** Samson Olusina Bamiwuye, Clifford Odimegwu

**Affiliations:** 1 Obafemi Awolowo University, Ile-Ife, Nigeria; 2 Visiting Scholar, University of the Witwatersrand, Johannesburg, South Africa; 3 Demography and Population Studies (DPS), University of the Witwatersrand, Johannesburg, South Africa

**Keywords:** Spousal violence, Sub-Sahara Africa, Household, Poverty-wealth, Domestic violence

## Abstract

**Introduction:**

Despite the threat of violence to the health and rights of women yet, for many years, there has been a dearth of nationally comparable data on domestic violence in sub-Saharan Africa. This paper examines whether women from poor households are more likely to experience violence from husband/partner than other women who are from middle or rich households.

**Method:**

Data for the study are derived from most recent DHS surveys of ever-married women age 15-49 in Cameroun(3,691), Kenya(4,336), Mozambique(5610), Nigeria (16,763), Zambia(3,010) and Zimbabwe(5,016) who participated in the questions on Domestic Violence Module. Bivariate analysis and Binary Logistic Regression Analysis are used to explore the linkage between household poverty-wealth and spousal violence while simultaneously controlling for confounding variables.

**Results:**

The overall prevalence of any form of violence (physical, sexual or emotional) ranges from 30.5% in Nigeria to 43.4% in Zimbabwe; 45.3% in Kenya; 45.5% in Mozambique; 53.9% in Zambia and 57.6% in Cameroun. Both bivariate and multivariate analyses show that in two of the six countries –Zambia and Mozambique, experience of violence is significantly higher among women from non-poor (rich) households than those from other households (poor and middle). For Zimbabwe and Kenya, women from poor households are more likely to have ever experienced spousal violence than those from non-poor households. In the remaining two countries- Nigeria and Cameroun, women from the middle class are more likely to have ever suffered abuse from husband/partner than those from the poor and rich households.

**Conclusion:**

Our results thus show that similar measurements of household poverty-wealth have produced varying relationships with respect to experience of spousal violence in six sub-Saharan African countries. In other words, experience of violence cuts across all household poverty-wealth statuses and therefore may not provide enough explanations on whether household-poverty necessarily serves to facilitate the ending of violence. These results suggest that eliminating violence against women in sub-Sahara Africa requires a comprehensive approach rather than addressing household poverty-wealth alone.

## Background

The United Nations General Assembly’s 1993 Declaration on the Elimination of Violence against Women broadly defined women violence as “any act of gender-based violence that results in or is likely to result in physical, sexual, or psychological harm or suffering to women, including threats of such acts, coercion or arbitrary deprivation of liberty whether occurring in public or private life [1].

Gender-Based Violence (GBV) have become a global issue that cuts across regional, social and cultural boundaries [2-6]. GBV not only poses a direct threat to women’s health, but also has adverse consequences for other aspects of women’s health and wellbeing and for the survival and wellbeing of children [1].

Home is supposed to be a secure environment, yet in many societies in sub-Saharan Africa, many women experience violence in diverse forms – physical, emotional psychological and sexual [7]. Much of this violence is perpetrated by women’s husbands or close partners [8]). Globally, research has shown that between 15 and 71 per cent of ever partnered women have been physically or sexually assaulted by an intimate partner at some time in their lives [9].

Across sub-Sahara Africa, reports of prevalence and incidence of GBV have been reported [10]. In Zambia, the problem of violence against women is worrisome and GBV is considered not an isolated problem or a side component of people’s life, but a widespread, tragic and daily issue that touches and impacts every Zambian in one way or the other [11]. In Zimbabwe, domestic violence has been described as a sensitive, harrowing community issue that affects 1 in 3 women with many women still finding themselves in a position where they are vulnerable to all forms of violence despite legislation to prevent domestic violence [12]. At least, 60% of the murder cases brought before the Zimbabwe High Court are a direct result of domestic violence [13]. Studies have also linked violence against women with negative child outcomes in Zimbabwe [12].

In Nigeria, the largest country in Africa, several studies have reported high prevalence of violence against women especially from spouse or intimate partner [5,6,14,15].

Despite the fact that violence against women in all forms has been acknowledged internationally as a threat not only to the health and rights of women but also to national development, yet, for many years, there has been a dearth of nationally comparable data such as the one from Demographic and Health Surveys on domestic violence [16]. Earlier research on domestic violence in the developing world contributed to a deeper awareness of the problem and since has been provoking research globally [2,17].

Different patterns of relationship between socio-demographic factors like age, education, age at marriage and spousal violence have been reported in the literature [8,18,19]. For example, while education is a protective factor in Bolivia, Kenya and Zimbabwe, it is a risk factor in Haiti [8]. While a study among pregnant women found a strong association between education and intimate partner violence [18], studies conducted in eight Southern African countries) show that high rates of domestic physical violence in all eight countries were conspicuously independent of education [19]. Studies have also indicated that younger women are more likely to be abused by partner/spouse than the older women [20] but women who marry young in Ghana and Uganda have higher rates of experience of spousal violence than those who marry at age 25 or older [21]. While there is no variation by residence in Ghana with women’s experience of violence, Ugandan women who live in rural areas have much higher rates of violence than those who live in urban areas [21].

Other factors that are often and consistently associated with spousal violence are partner’s alcohol use [1,18]; acceptance of wife beating as justified by men and women [22,23] and history of respondent’s father beating her mother [1,23,24]. Widespread acceptance of wife beating as justified is found to be consistent with a high prevalence of violence [23].

Literature on association between poverty-wealth and spousal violence is relatively scanty especially in sub-Sahara Africa. The few available studies have focused on countries in Latin America/Caribbean and South/Southern Asia [1,16]. For example [1] used only one country in sub-Saharan Africa in a Multi-country study of domestic violence in nine countries. In three Asian countries [16] examined the link between poverty and violence, their emphasis was not so much on poverty-violence relationship but how poverty interlinked with violence to affect some selected reproductive health outcomes. Even in the few available studies literature on poverty-violence relationship, findings have been inconsistent [16,25]. Research has also shown that experience of spousal violence varies nonlinearly with household wealth in Ghana, with violence at highest among women in households in the middle wealth quintiles while in Uganda prevalence of experience of violence declines by household wealth [21]. In Uganda, prevalence of experience of violence among men and women in the highest wealth quintile is less than half of the level among those in the lowest wealth quintile [21]. In Nigeria, low socioeconomic status is a risk factor for experiencing abuse among pregnant women [20].

While in a study conducted in India, there is an inverse relationship between socioeconomic status and partner violence [26], in Mexico, severe physical violence was found to be significantly lower in high socioeconomic households compared with low socioeconomic households [27]. In Turkey, though income was found to be significant with partner violence, but the direction of the relationship is not clear [28].

A study of three countries found that highest asset quintile significantly associate with lower violence when compared to the poorest socioeconomic group [1] and in India, higher household income was significantly associated with lower physical violence [29]. In Nigeria, by contrast, higher income was significantly associated with higher physical violence [6].

Although, there was a substantially significant association between spousal violence and the wealth index in three Asian countries [16], the direction and strength of the association varied between countries. In Cambodia, for example, the likelihood of spousal violence declines with wealth only from the first to the third quintile and does not vary at all between the top three quintiles. In the Dominican Republic, by contrast, only women in the wealthiest quintile have a significantly lower likelihood of experiencing spousal violence and in Haiti, women in the third quintile were the most likely to have experienced spousal violence (16).

The inconsistent pattern of association between spousal violence and household poverty-wealth as evidenced in the existing literature is probably due to methodological issues with respect to differences in measurement of wealth status/socioeconomic status and variations in measurement of domestic violence. For example, some data were collected before the development of the domestic violence module; such data used a single-question threshold approach in which a respondent is asked whether she has ever experienced violence. The limitation here is that what constitute violence may differ from culture to culture. Secondly, not all countries for which domestic violence data were collected by DHS surveys have used the module [1]. Rather than using a single-question threshold approach to measure spousal violence, as in the case of Eqypt, India, Peru and Zambia [1] our paper adopts the modified CTS approach as embodied in the DHS domestic violence module which consists of 15 acts of physical and sexual violence out of the 19 acts in the original CTS [1,30]. Thus we examine whether associations between household poverty and spousal violence will be consistent using similar measurements and nationally representative and comparable datasets. Specifically, we examine in six sub-Saharan African countries whether women from poor households are more likely to be abused by their spouse than other women who are from the middle or rich households.

## Methods

### Data source and sample

Data from the most recent survey was obtained for six countries from MEASURE DHS for the analyses in this study: the 2011 Cameroun DHS, the 2008-9 Kenya DHS; the 2011 Mozambique DHS; the 2008 Nigeria DHS; the 2007 Zambia DHS and the 2010-2011 Zimbabwe DHS. Each of the surveys collected nationally representative data on domestic violence for women in reproductive age group 15-49. For the purposes of this paper, the sample was limited to ever married women who participated in the domestic violence module. Specially constructed weights were used to ensure that the domestic violence subsample was nationally representative. This is because only one woman per household received the domestic violence module. The DHS sampling weight variable “d005” corrects for oversampling and under-sampling using the Stata command “gen wt = d005/1000000”. With this constraint, a weighted sample of 3691 ever married women in Cameroun; 4336 in Kenya, 5610 in Mozambique; 16,763 in Nigeria, 3010 ever-married women in Zambia and 5016 in Zimbabwe constitute our samples in the study.

## Ethical considerations

The survey procedures and instruments used for the study were ethically approved by the Ethics Committee of the ICF Macro International, Inc, Calverton, Maryland and by the National Ethics Committee of each country. Data collections were done after obtaining the informed consents from all the respondents, and participants identifiers were removed to ensure confidentiality. We obtained ethical permission for use of all the datasets in the study from ICF Macro.

### Measurement of violence in DHS domestic violence module

The DHS violence module obtained information from ever-married women on violence by spouses and others, and from never-married women on violence by anyone, including boyfriends. Spousal/partner violence was measured in more detail than violence by other perpetrators through use of a modified Conflict Tactics Scale (CTS) [31]. The original CTS was developed by Murray Straus in the 1970s and it consists of a series of individuals questions on specific acts of violence [1]. Specifically, spousal violence by the husband/partner for currently married women and the most recent husband/partner for formerly married women was measured by asking all ever-married women the following set of questions:

Does (did) your (last) husband/partner ever:

a) Say or do something to humiliate you in front of others?

b) Threaten to hurt or harm you or someone close to you?

c) Insult you or make you feel bad about yourself?

Does (did) your (last) husband/partner ever do any of the following things to you?

d) Push you, shake you, or throw something at you?

e) Slap you?

f) Twist your arm or pull your hair?

g) Punch you with his fist or with something that could hurt you?

h) Kick you, drag you, or beat you up?

i) Try to choke you or burn you on purpose?

j) Threaten or attack you with a knife, gun, or any other weapon?

k) Physically force you to have sexual intercourse with him even when you did not want to?

l) Force you to perform any sexual acts you did not want to?

A “yes” answer to one or more of items (a) to (c) above constitutes evidence of emotional violence, a “yes” answer to one or more of items (d) to (j) constitutes evidence of physical violence, and a “yes” answer to items (k) or (l) constitutes evidence of sexual violence [2]. DHS defines “less severe violence” as a “yes” to one or more of items “d” to “g”, and “severe violence” as a “yes” to one or more of items “h” to “j” [2].

### Explanatory variable –household poverty-wealth

We estimate household poverty-wealth by using wealth index developed by the DHS and which has been tested and found consistent in a large number of countries with regard to inequities in household income [32,33]. Wealth index is a quintile with those that are poorest at the first quintile and richest at the last quintile. We categorize household poverty-wealth into poor, middle and rich to conform to African standard of explaining household poverty-wealth status where there is a high inequality in income distribution. This provides an important tool for understanding the status of the ‘average’ and provides policy makers with a more balanced assessment of development [34].

### Outcome variable –spousal violence

Outcome variable is spousal violence, measured in three dimensions in this study:

i) Ever experienced physical violence ii) ever experienced sexual violence and iii) ever experienced emotional violence

A number of selected background characteristics, and other variables that have been found in earlier studies to be significantly associated with spousal violence are included in the analysis. These variables are respondent’s age, level of education, residence, alcoholic intake, respondent’s history of violence [1,14].

### Data analysis

We tabulate forms and severity of violence in order to measure prevalence and tabulate according to household poverty-wealth status in order to examine patterns of associations between household poverty-wealth and spousal violence using the Chi Square statistic. At the multivariate level, we presents four models of binary logistic regression analysis to obtain the odds of ever experience of spousal physical violence and any form of violence (physical, sexual or emotional) by selected variables. The first model presents the unadjusted Odd Ratios (ORs) of household poverty-wealth on ever-experience of spousal violence. The second model presents the adjusted ORs of household poverty-wealth on ever experience of spousal violence controlling for socio-demographic variables like age, education, marital status (current and former), age at marriage and current work status. The third model relates variables that have been found consistent in previous studies to associate with ever–experience of spousal violence. These variables are attitude to spousal violence – a binary variable on whether or not husband is justified in beating his wife on any condition; husband/partner alcohol intake; and history of violence –whether respondent father ever beat mother. The fourth model is a full model showing the adjusted odds of ever experiencing spousal violence controlling for socio-demographic variables; partner alcohol intake, history of violence, and attitude to spousal violence. We used the STATA software version 12 for the analyses.

## Results and discussion

### Background characteristics

Table 1 provides percent distribution of respondents in all the six countries by background characteristics and household poverty-wealth status. The percentage age distribution of the respondents shows that Mozambique has the highest proportion of people below age 25 (30.1%) with a mean age of 30.7 while Kenya has the least proportion of ever married women in the youngest age group (22.6%) with a mean age of 32. Thus the mean age of ever married women in the six countries ranges from 30.7 to 32.0 years.

**Table 1:** Percent distribution of respondents by background characteristics and household poverty-wealth status

	**Cameroun**	**Kenya**	**Mozambique**	**Nigeria**	**Zambia**	**Zimbabwe**
	**% (N)**	**% (N)**	**% (N)**	**% (N)**	**% (N)**	**% (N)**
**Age**						
less than 25	27.8 (1025)	22.6 (981)	30.1 (1689)	23.4 (3923)	25.3 (989)	27.3(1367)
25-34	37.7 (1392)	40.2 (1744)	35.6 (1997)	39.2 (6564)	41.8 (1634)	39.7 (1993)
35+	34.5 (1274)	37.2 (1611)	34.3 (1294)	47.4 (6276)	32.9 (1287)	33.0 (1656)
**Mean**	30.9	32.0	30.7	31.3	31.1	30.7
**Age at marriage**						
less than 18	54.0 (1994)	38.4 (1667)	51.8 (2909)	56.8 (9526)	54.1 (2114)	37.2 (1864)
18 -24	36.8 (1359)	52.4 (2271)	39.1 (2192)	32.6 (5456)	41.6 (1628)	53.7 (2696)
25+	4.3 (338)	9.2 (398)	9.1 (509)	10.4(1781)	4.3 (168)	9.1 (456)
**Mean children ever born**	3.6	3.7	3.5	4.0	4.0	2.7
**Education**						
None	25.1 (927)	11.8 (513)	36.2 (2032)	46.7 (7831)	12.7 (498)	3.0 (150)
Primary	37.9 (1398)	59.8 (2592)	50.8 (2851)	22.5 (3775)	60.0 (2345)	32.5 (1629)
Secondary +	37.0 (1366)	28.4 (1230)	13.0 (727)	30.8 (5157)	27.3 (1067)	64.5 (3237)
**Percent Currently working**						
	71.6 (2642)	65.6 (2846)	43.7 (2449)	67.3 (11286)	53.9 (2109)	40.3 (2023)
**Household characteristics**						
**Residence**						
Urban	50.4 (1858)	23.6 (1022)	30.7 (1722)	31.6 (5289)	37.1 (1449)	33.8 (1696)
Rural	49.6 (1833)	76.4 (3314)	69.3 (3888)	68.4 (11474)	62.9 (2461)	66.2 (3320)
**Household poverty-wealth**						
Poor	37.2 (1372)	40.3 (2261)	40.3 (2261)	44.3 (7427)	39.1 (1528)	38.0 (1906)
Middle	19.2 (710)	21.2 (1189)	21.2 (1189)	18.6 (3111)	19.5 (765)	19.6 (981)
Rich	43.6 (1609)	38.5 (2160)	38.5 (2160)	37.1 (6225)	41.4 (1617)	42.4 (2129)
**ALL**	3691	4336	5610	16763	3910	5016

At least half of the respondents have been married before 18 in four of the six countries – Cameroun(54.0%); Mozambique (51.8%); Nigeria (56.8%) and Zambia (54,1%). The mean total number of children ever born ranges from 2.7 in Zimbabwe to 4.0 in Nigeria and Zambia. Nearly half of the women (46.7%) in Nigeria and 3.0% in Zimbabwe reported no formal education. At least 1 in 4 women from Cameroun and close to 2 in 5 women from Mozambique (36.2%) have no formal education. Sixty five percent of women from Zimbabwe; 37% from Cameroun and 31% from Nigeria have acquired secondary education or higher.

An examination of economic activities measured by current work status, shows that more than 3 in 5 of our respondents in 3 countries, more than half in one country and less than half in two countries were currently engaged in one economic activity or the other.

In terms of household characteristics, with the exception of Cameroun, more than 3 in 5 women live in the rural areas in all the countries. The distribution according to household poverty-wealth status reveals that at least 40% of our sample are from poor households in 3 countries – Kenya (40.3%); Mozambique (40.3%) and Nigeria (44.3%). In three countries, at least 2 in 5 women, as classified by household assets, are from rich households – Cameroun (43.6%); Zimbabwe (41.4%) and Zambia (42.4%). Thus the distribution of respondents by household poverty-wealth status is roughly the same in all the six countries.

### Household poverty-wealth status and experience of spousal violence

Table 2 shows the percent distribution of ever-married women by experience of spousal violence according to household poverty-wealth status. The prevalence of spousal violence (physical, sexual or emotional) is 57.6% in Cameroun and this is highest among women from middle class of household poverty-wealth status (61.3%) compared with those from poor households (55.4%) and rich households (57.9%). The overall prevalence of physical violence is 45.4%; also highest among the women from rich households (46.7%) and least among women from poor households (43.1%). Similarly, for ever married women in Cameroun, the prevalence of sexual violence is 16.0%; 19.4% among women from rich households, 14.6% and 15.8% among women from poor and middle household poverty-wealth statuses respectively.

**Table 2:** Percent distribution of ever-married women by experience of spousal violence according to household poverty-wealth status

	**Cameroun household poverty-wealth status**					
	**Poor**	**Middle**	**Rich**	**All**	**N**	**Sig**
**Forms of violence**						
any physical violence	43.1	46.7	45.4	44.8	1653	ns
any sexual violence	14.6	19.3	15.8	16.0	592	ns
any emotional violence	41.2	44.7	41.2	41.9	1546	ns
physical and sexual	11.3	15.0	13.0	12.8	471	ns
physical, sexual or emotional	55.4	61.3	57.9	57.6	2128	ns
**Severity of physical violence**						
less severe violence	42.7	45.7	45.1	44.3	1636	ns
severe violence	10.5	14.1	13.3	12.4	458	ns
	**Kenya household poverty-wealth status**					
	**Poor**	**Middle**	**Rich**	**All**	**N**	**Sig**
**Forms of violence**						
any physical violence	42.4	38.6	31.8	37.0	1603	<0.01
any sexual violence	15.6	15.9	12.8	14.4	626	ns
any emotional violence	30.7	29.5	28.5	29.5	1279	ns
physical and sexual	13.4	14.5	10.1	12.2	528	ns
physical, sexual or emotional	49.9	45.7	41.3	45.3	1964	<0.01
**Severity of physical violence**						
less severe violence	41.4	38.1	31.5	36.4	1578	<0.01
severe violence	19.7	14.9	15.6	16.9	735	ns
	**Mozambique household poverty-wealth status**					
	**Poor**	**Middle**	**Rich**	**All**	**N**	
**Forms of violence**						
any physical violence	29.3	30.3	34.4	31.5	1765	<0.05
any sexual violence	7.0	7.2	9.2	7.8	442	ns
any emotional violence	30.5	33.5	37.5	33.8	1896	<0.01
physical or sexual	5.8	5.5	7.1	6.2	350	ns
physical, sexual or emotional	41.3	44.9	50.1	45.5	2550	p < 0.01
**Severity of physical violence**						
less severe violence	28.6	28.7	33.4	30.5	1710	p < 0.05
severe violence	9.0	11.5	13.1	11.1	623	p < 0.05
	**Zambia household poverty-wealth status**					
	**Poor**	**Middle**	**Rich**	**All**	**N**	**Sig**
**Forms of violence**						
any physical violence	42.7	45.2	50.7	46.5	1817	P < 0.01
any sexual violence	13.6	16.2	19.8	16.7	1671	P < 0.01
any emotional violence	21.4	22.8	30.8	25.6	1001	
physical and sexual	10.9	13.6	16.5	13.7	1373	p < 0.01
physical, sexual or emotional	49.01	52.3	59.2	53.9	2106	p < 0.01
**Severity of physical violence**						
less severe violence	42.4	44.6	50	46	1798	p < 0.01
severe violence	12.6	13.7	16.4	14.4	563	p < 0.05
	**Zimbabwe household poverty-wealth status**					
		**Poor**	**Middle**	**Rich**	**All**	**N**
**Forms of violence**						
any physical violence	32.5	30.2	24.9	28.8	1445	p < 0.01
any sexual violence	16.1	16	14.8	15.5	779	ns
any emotional violence	28.4	27.1	24.4	26.5	1327	P < 0.05
physical or sexual	9.6	10	8.5	9.2	461	ns
physical, sexual or emotional	47.3	45.6	38.9	43.4	2177	p < 0.01
**Severity of physical violence**						
less severe violence	31.5	29.4	23.7	27.8	1394	p < 0.01
severe violence	10.2	10.5	8.9	9.7	486	ns
	**Nigeria household poverty-wealth status**					
	**Poor**	**Middle**	**Rich**	**All**	**N**	
**Forms of violence**						
any physical violence	13.8	21.6	19.8	17.5	2929	p < 0.01
any sexual violence	3.2	5.6	3.8	3.9	652	p < 0.01
any emotional violence	24.4	27.4	20.8	23.6		p < 0.01
physical or sexual	2.4	4.8	3.0	3.1	510	p < 0.01
physical, sexual or emotional	29.5	35.2	29.4	30.5	5117	p < 0.01
**Severity of physical violence**						
less severe violence	13.5	21.0	19.2	17.0	2851	p < 0.01
severe violence	4.8	8.7	7.7	6.6	1105	p < 0.01

*ns* not significant.

In Kenya, the prevalence of spousal physical violence among ever-married women is 37.0%; for sexual violence 14.4% while for emotional violence is 29.5%. The prevalence of any of the three forms of spousal violence is 45.3%. In relation to household poverty-wealth status, the prevalence of spousal violence is generally higher among the poor than among middle and rich households.

In Mozambique, the emotional violence is the commonest with prevalence of 33.8% against sexual violence (7.8%) and physical violence (31.5%). However the direction of relationship between household poverty-wealth and experience of violence contrasts with that of Kenya, with highest prevalence among the rich for all forms of violence compared with their counterparts from the middle and poor households.

In Nigeria, women from the middle class of household poverty-wealth are more likely to report ever-experience of violence from husband/partner when compared with other women who are from poor households or who are from rich households. Specifically, the prevalence of spousal physical violence generally is 17.5% and in terms of household poverty-wealth status comprises 13.8% of ever-married women from poor household, 21.6% from middle and 19.8% from the rich households. The prevalence of spousal sexual violence is 3.9% and similarly the highest among women in the middle class status (5.6%). Generally, for any form of spousal violence, the prevalence is 30.5% for ever-married women in Nigeria and this is consistently higher among women from the middle class when compared with women from poor and rich households.

In Zimbabwe, the percentage of ever married women who have ever experienced any act of physical violence is 28.8% and in Zambia, it is 46.5%. The percentage of ever married women that experienced any sexual violence is 15.5% in Zimbabwe and 16.7% in Zambia. While 26.5% reported experience of emotional violence in Zimbabwe, 25.6% have ever experienced emotional violence in Zambia. Sexual violence is the least reported of all the three forms of spousal violence in all the six countries. There are proportions of ever married women who have experienced a combination of physical and sexual violence or who have experienced either physical violence or sexual violence or both. The proportion of ever-married women who have experienced any form of spousal violence in Zambia is 49.5%, and this is higher than the women in Zimbabwe (35.2%). In contrast to Zambian women, the prevalence of any form of spousal violence is higher among women from poor household than those from middle or from rich households. For instance, 32.5% of women from poor households compared with 30.5% and 24.9% from middle and rich households respectively are more likely to report ever-experience of physical violence.

Prevalence of severity of spousal physical violence is the highest among women in the middle class in three of the six countries – Cameroun (14.1%); Zimbabwe (10.5%) and Nigeria (8.7%). The prevalence is highest among women from rich households in Zambia (16.4%) and Mozambique (13.1%) and higher among women from poor households in only one country – Kenya (13.1%).

### Multivariate analysis

Tables 3 and 4 present four models of logistic regression analysis of experiencing physical violence and any forms of spousal violence (physical, emotional or sexual).

**Table 3:** Odds of ever-experience of any act of physical spousal violence among ever-married women

**Explanatory variable**	**Model 1**^**a**^	**Model2**^**b**^	**Model3**^**c**^	**Model4**^**d**^
**Household poverty-wealth**		Cameroun		
**Poor**	RC	RC	RC	RC
**Middle**	1.16	1.10	1.09	1.22
**Rich**	1.10	1.07	1.10	1.22
	Kenya			
**Poor**	RC	RC	RC	RC
**Middle**	0.86	0.89	0.86	0.86
**Rich**	0.63*	0.77*	0.78	0.91
	Mozambique			
**Poor**	RC	RC	RC	RC
**Middle**	1.05	1.07	1.05	1.08
**Rich**	1.26*	1.27*	1.24*	1.27*
	Nigeria			
**Poor**	RC	RC	RC	RC
**Middle**	1.72**	1.28**	1.52**	1.33**
**Rich**	1.55**	0.94	1.45**	1.21*
	Zambia			
**Poor**	RC	RC	RC	RC
**Middle**	1.11	1.12		1.15
**Rich**	1.38*	1.44*		1.45*
	Zimbabwe			
**Poor**	RC	RC	RC	RC
**Middle**	0.90	0.89	0.96	0.93
**Rich**	0.69**	0.75**	0.82*	0.81*

^a^unadjusted ORs; ^b^ORs adjusted for age, education age at marriage, current work status, and marital status (not included).

^c^ORs adjusted for history of violence, attitude towards violence and partner alcoholic intake (variables not included).

^d^ORs adjusted for variables in (b) and (c) above (variables not included in the Table).

**p < 0.01 *p < 0.05.

**Table 4:** Odds of ever-experience of any form of spousal violence among ever-married women

	**Model 1**^**a**^	**Model2**^**b**^	**Model 3**^**c**^	**Model 4**^**d**^
**Household poverty-wealth**				
		**Cameroun**		
**Poor**	RC	RC	RC	RC
**Middle**	1.27	1.16	1.21	1.21
**Rich**	1.11	1.01	1.11	1.16
		**Kenya**		
**Poor**	RC	RC	RC	RC
**Middle**	0.84	0.85	0.84	0.84
**Rich**	0.702**	0.84	0.86	0.98
		**Mozambique**		
**Poor**	RC	RC	RC	RC
**Middle**	1.16	1.18	1.17	1.2
**Rich**	1.43**	1.41**	1.43**	1.44**
		**Nigeria**		
**Poor**	RC	RC	RC	RC
**Middle**	1.30**	1.14**	1.17*	1.14*
**Rich**	0.99	0.83**	0.95	0.97
		**Zambia**		
**Poor**	RC	RC	RC	RC
**Middle**	1.14	1.16	1.17	1.18
**Rich**	1.51**	1.53**	1.58**	1.54**
		**Zimbabwe**		
**Poor**	RC	RC	RC	RC
**Middle**	0.93	0.92	0.96	0.93
**Rich**	0.71**	0.74**	082*	0.81*

^a^unadjusted ORs; ^b^ORs adjusted for age, education age at marriage, current work status, and marital status (not included).

^c^ORs adjusted for history of violence, attitude towards violence and partner alcoholic intake (variables not included).

^d^ORs adjusted for variables in (b) and (c) above (variables not included in the Table).

**p < 0.01 *p < 0.05.

In the first Model, the unadjusted odd of experiencing physical or any form of spousal violence (physical, sexual or emotional) is the highest for women of middle household poverty-wealth status in Cameroun and Nigeria; highest for women from rich household in Zambia and Mozambique, and highest among women from poor households. Specifically, women from poor households in Kenya and Zimbabwe are significantly more likely to experience spousal violence than women from middle and rich households. In contrast, women from rich households in Mozambique and Zambia are significantly more likely to be abused than women from middle or poor households. In Cameroun the relationship between household poverty-wealth and experience of spousal violence is not significant but in Nigeria women from middle households of household poverty-wealth status are significantly more likely to report ever-experience of spousal violence, whether physical violence only or any physical, sexual or emotional violence. This result is consistent with those obtained at the bivariate level of analysis.

In Model 2, the odds of ever experience of physical spousal violence increases from 0.63 to 0.77 among the women from rich households in Kenya; from 1.38 to 1.44 in Zambia; 1.26 to 1.27 in Nigeria; and from 0.69 to 0.75 in Zimbabwe. In Model 3, the inclusion of history of violence, partner alcoholic intake and attitude to violence to our explanatory variable did not change the direction of the relationship between household poverty-wealth and spousal violence.

In Model 4, women from poor households in Cameroun are less likely to report ever being abused physically or suffer physical, sexual or emotional violence from their spouse or partner when compared with those from middle or rich households, although this relationship is not statistically significant. In Kenya, the adjusted odd of ever-experience of violence remains the same (aOR = 0.86) for women from middle class, but increases for women from rich households (aOR = 0.91). However, the direction of the relationship still remains the same. In Nigeria, the adjusted OR of ever experience of spousal violence is consistently higher among the women from middle household poverty-wealth status than other women in all the Models. In Zambia, the adjusted odd of ever experience of spousal violence by ever-married women from rich households is consistently highest in all the Models. In Zimbabwe, women from poor households are significantly more likely to report ever experience of spousal violence than other women and this is consistent in all the Models.

While the relationship between household poverty-wealth and ever-experience of spousal violence is consistent for each country, the direction of the relationship differs across countries.

## Discussion

In this study we used the most recent DHS data from six sub-Saharan African countries who participated in the domestic violence module to examine the relevance of household poverty-wealth in explaining experience of spousal violence among ever-married women. The distribution of respondents by household poverty-wealth status is roughly the same in all the six countries. The prevalence of spousal physical violence among ever married women ranges from 17.5% in Nigeria to 46.5% in Zambia. The prevalence of spousal sexual violence is as low as 3.9% in Nigeria to as high as 16.7% in Zambia while the prevalence of spousal emotional violence ranges from 23.6% in Nigeria to 41.9% in Cameroun. Previous studies have also noted the high prevalence of physical and sexual violence in Zambia compared to many other countries [1,10].

Findings from two of the six countries –Zambia and Mozambique also show that experience of violence is significantly higher among women from non-poor (rich) households than those from other households (poor and middle). Although this result deviates from the expected, similar findings were reported in the past [1,19]. However for Zimbabwe and Kenya, women from poor households are more likely to have ever experienced spousal violence than those from non-poor households. This result is in the expected direction assumed by the literature [17,35]. In the remaining two countries- Nigeria and Cameroun, women from the middle class are more likely to have ever suffered abuse from husband/partner than those from the poor and rich households. The relationship between household poverty-wealth and experience of violence among ever-married women is not statistically significant for only one country (Cameroun).

The results of the multivariate analysis model are consistent with those obtained at the bivariate level of analyses for all the six countries even after adjusting for some socio-demographic characteristics like age, education, age at marriage, marital status (currently and formerly married) and current work status. The results are also consistent after adjusting for the effects of some variables the literature consistently found to be associated with spousal violence (history of violence, attitude to violence, and husband/partner alcoholic intake). Our results thus show that similar measurements of household poverty-wealth have produced varying relationships with respect to experience of spousal violence in six sub-Saharan African countries. These results reveal that experience of violence cuts across all women whether they are from poor, middle or rich households.

## Conclusion

The primary strength of this study is that it is based on fully comparable and nationally representative data from six countries of sub-Saharan Africa drawn on the basis of geographical location. This is because this type of comparison has rarely been done in sub-Saharan Africa in the field of spousal violence at it relates to relevance of household poverty-wealth status.

Our results contribute towards a better understanding of the link between household poverty-wealth and experience of domestic violence among women in sub-Saharan Africa by showing that experience of violence cuts across women from different households, poor, middle or rich.

While experience of violence is significantly higher among women from rich households, than those from the middle and poor households in Zambia and Mozambique, it is not so for Zimbabwe and Kenya where more women from poor households experienced spousal violence than those from middle and rich households. In Nigeria, and Cameroun, women from the middle class are more likely to have ever suffered abuse from husband/partner than those from the poor and rich households. The findings above compared favourably with study of three Asian Countries – Cambodia, Dominican Republic and Haiti (1). Both bivariate and multivariate analyses did not reveal a consistent association between household poverty-wealth and women experience of domestic violence. These results suggest that eliminating violence against women requires a comprehensive approach rather than addressing poverty alone. This is an important implication for any policy and programmes aimed at reducing domestic violence levels in sub-Saharan Africa.

Despite the usefulness of the findings arising from this study, there are a number of limitations, such as the cross-sectional nature of the data which cautions us from making any causal inference between household poverty-wealth and violence and the fact that the study relies mainly on women’s report of violence suffered from their partners/spouse.

## Abbreviations

CTS: Conflict tactics scale; DHS: Demographic and health survey; GBV: Gender-based violence.

## Competing interests

The authors declare that they have no competing interests.

## Authors’ contributions

Both authors were responsible for the design and conduct of the study; analyses were done by SO; interpretation of findings and drafting of the manuscript were done by both authors. The authors read and approved the final content of the manuscript before submission for publication.

## Authors’ information

SO is a Senior Lecturer and former Acting Head of Department of Demography and Social Statistics, Obafemi Awolowo University, Ile-Ife, Nigeria and a Visiting Scholar, Demography and Population Studies Programme, University of the Witwatersrand, Johannesburg. C is a Professor and Head, Demography and Population Studies Programme, University of the Witwatersrand, Johannesburg, South Africa.
